# Assessing the relationship between low-calorie sweetener use and quality of life measures in adults with type 1 diabetes

**DOI:** 10.1186/s12902-023-01399-y

**Published:** 2023-07-11

**Authors:** Halis K Akturk, Janet Snell-Bergeon, Kagan E Karakus, Viral N Shah

**Affiliations:** Barbara Davis Center for Diabetes, University of Colorado, Aurora, CO USA

**Keywords:** Type 1 diabetes, Quality of life, Low-calorie sweeteners

## Abstract

**Background:**

To evaluate use of low-calorie sweeteners (LCS) among adults with type 1 diabetes (T1D) and its impact on quality of life (QOL).

**Methods:**

In this single center, cross-sectional survey study with 532 adults with T1D, Food related QOL (FRQOL), LCS specific questionnaire (LCSSQ), Diabetes Self-Management Questionnaire (DSMQ), Food Frequency Questionnaire (FFQ), Audit of Diabetes-Dependent QOL (AddQOL), Type 1 Diabetes and Life (T1DAL) questionnaires were administered through RedCAP, a secure, HIPAA-compliant web-based application. Demographics and scores of adults who used LCS in last month (recent users) and others (non-users) were compared. Results were adjusted for age, sex, diabetes duration and other parameters.

**Results:**

Of 532 participants (mean age 36 ± 13, 69% female), 99% heard LCS before, 68% used them in the last month, 73% reported better glucose control with LCS use and 63% reported no health concerns about LCS use. Recent LCS users were older and had a longer diabetes duration and more complications (hypertension, or any complication) than non-users. However, A1c, AddQOL, T1DAL, FRQOL scores did not differ significantly between recent LCS users and non-users. DSMQ scores, DSMQ management, diet, health care scores did not differ between two groups; however, recent LCS users had lower physical activity score than non-users (p = 0.001).

**Conclusions:**

Most of the adults with T1D have used LCS and perceived that LCS use improved their QOL and glycemic control; however, these were not verified with questionnaires. There was no difference in QOL questionnaires except DSMQ physical activity between recent LCS users and not users with T1D. However, more patients in need to increase their QOL may be using LCS; therefore, associations between the exposure and outcome can be bi-directional.

## Background

Less than a third of adults with type 1 diabetes (T1D) in the US meet American Diabetes Association goals for glycemic control (i.e., A1c < 7%) [1]. One of the major reasons for uncontrolled diabetes is postprandial hyperglycemia, which is caused by a mismatch between insulin dose or timing and carbohydrate intake [2]. The consumption of higher amounts of carbohydrates has a direct effect on postprandial glycemia. Randomized controlled trials have shown that diets with low glycemic index and low glycemic load improve glycemia in adults with diabetes [3]. Guidelines also recommend reducing the consumption of refined carbohydrates and sugar-sweetened beverages in diabetes management [4]. In this respect, low-calorie sweeteners (LCS) offer an option to replace higher caloric sugar ingredients and provide a sweeter taste in unit weight [5, 6]. The use of LCS may reduce carbohydrate intake, improve glycemic control, and decrease weight [7]. However, their association with quality of life (QOL) has not been well studied in people with T1D. Therefore, we investigated the use of low-calorie sweeteners among adults with type 1 diabetes and its impact on quality of life.

## Methods

This is an investigator-initiated single-center, cross-sectional survey study on a clinic-based cohort of 532 adults with T1D. In this study, adults with T1D at the Barbara Davis Center for Diabetes received validated questionnaires about the use of LCS and QOL through RedCap, a secure HIPAA-compliant web-based application. The questionnaire was distributed online through RedCap between January 1, 2021, to July 1, 2021 and individuals responded to the questionnaires using their personal devices. The LCS was defined as sweeteners that contain few to no calories but have a higher intensity of sweetness per gram than sweeteners with calories-like table sugar, fruit juice concentrates, and corn syrups. Other common names for LCS such as non-nutritive sweeteners, artificial sweeteners, sugar substitutes, and high-intensity sweeteners were also provided for participants. The most used brands were also mentioned in the introduction part of the survey. Colorado Multiple Institutional Review Board approved this study under the exempt category. Data is available upon request from the corresponding author.

To assess the QOL, several questionnaires were used: Food related QOL (FRQOL) [8], LCS-specific questionnaire (LCSSQ), Diabetes Self-Management Questionnaire (DSMQ) [9], Harvard Willett Food Frequency Questionnaire (FFQ) [10], Audit of Diabetes-Dependent QOL (AddQOL) [11], Type 1 Diabetes and Life (T1DAL) [12].

The FRQOL is used to assess the QOL aspects related to eating behaviors, adapted from FRQOL used in inflammatory bowel disease. The LCSSQ was to assess current LCS use and knowledge among study participants. The DSMQ has overall and subscale scores for dietary control, physical activity, healthcare, and glucose management. Higher scores indicate better management. The FFQ was to assess average food intake over the past year with a focus on LCS use. The AddQOL was to assess how diabetes affects QOL, with a more negative score indicating more of an effect of T1D on QOL. The T1DAL examines the effects of T1D on QOL aspects that are specific to the life stage.

Overall characteristics of study participants were examined, categorical variables were presented as absolute numbers and percentages, and continuous variables were presented as mean and standard deviation (SD). Participants were divided into two groups based on usage of LCS in the last month, people who used LCS in the last month were defined as recent LCS users, and others as LCS non-users. Differences in demographics, glucose control and management, and QOL metrics were compared between the two groups. A chi-square test was performed to compare categorical variables, and a student t-test was performed for continuous variables. In a linear model adjusted for age, sex, diabetes duration, and complications, A1c, DSMQ scores, DSMQ diet scores, and DSMQ physical activity scores were compared by LCS use. In another model adjusted for age, sex, diabetes duration, daily breakfast, eating outside of the home, trying to lose weight, and complications; AddQOL, T1DAL, and FRQOL scores were compared by LCS use. The statistical significance threshold was determined as a two-tailed P-value < 0.05.

## Results

Out of 532 adults with T1D, the age was 36 ± 13, 69% were female, 92% were non-Hispanic White, 42% were college graduates, 26% had a professional degree and 82% had private insurance. Table 1 shows the baseline characteristics of the recent LCS users and non-users with T1D.

**Table 1:** Baseline characteristics of recent LCS users and non-users with T1D

Characteristic	Recent LCS use (n = 360)	No recent LCS use (n = 172)	P-value
Age (years)	37 ± 13	33 ± 12	0.0062
Diabetes duration (years)	22 ± 13	19 ± 11	0.0095
Sex (n[%]) male	112 (31.1)	53 (30.8)	0.9447
HbA1c, %	7.2 ± 1.2	7.4 ± 1.6	0.2644
Diabetic ketoacidosis (n[%])	10 (2.78)	9 (5.23)	0.1657
Race (n[%])			0.4455
American Indian/Alaska Native	2 (0.56)	1 (0.40)	
Asian Black Multiracial Native Hawaiian Other White	2 (0.56) 3 (0.83) 16 (4.44) 1 (0.28) 2 (0.56) 334 (92.8)	2 (0.79) 2 (1.16) 9 (5.23) 0 (0) 0 (0) 158 (91.9)	
Ethnicity (n[%])			0.0380
Hispanic Not Hispanic	21 (5.83) 339 (94.17)	19 (11.05) 153 (88.95)	
Education (n[%])			0.1656
Less than High School High School Graduate Some College College Graduate Graduate or Professional	4 (1.11) 20 (5.56) 89 (24.72) 162 (45.00) 85 (23.61)	2 (1.16) 14 (8.14) 38 (22.09) 63 (36.63) 55 (31.98)	
Insurance (n[%])			0.1525
Private Insurance Public Insurance (Medicaid, Tricare, Medicare) or uninsured	303 (84.17) 57 (15.83)	136 (79.07) 36 (20.93)	
Hypertension n(%)	97 (26.94)	26 (15.12)	0.0019
High Cholesterol n(%)	127 (35.28)	47 (27.33)	0.0651
Diabetic Kidney Disease n(%)	24 (6.67)	8 (4.65)	0.3501
Diabetic Retinopathy n(%)	51 (14.17)	17 (9.88)	0.1581
Diabetic Neuropathy n(%)	38 (10.56)	15 (8.72)	0.5087
Coronary Artery Disease n(%)	4 (1.11)	1 (0.58)	0.5366
Any Complication n(%)	194 (53.89)	73 (42.44)	0.0134

FFQ revealed that 93% of the participants were eating 2–3 meals a day, 54% were cooking meals at home daily, 31% were eating meals outside 2–3 times a week, 53% were eating breakfast every day, 65% were eating lunch every day, 91% were eating dinner every day and 73% were snacking 1–2 times in a day. Out of the participants, 75% of them have tried to cut down their sugar intake in the last year. The main purposes were to have better glycemic control (81%), to lose weight (60%), and to increase the QOL (33%).

Almost all (99%) of them heard of LCS, 90% of them have used it before and 68% of them have used it in the last month. Aspartame was the most used LCS (41%), followed by Stevia (31%), sucralose (17%), and saccharin (8%). The LCS was used mostly in drinks (95%). The majority (73%) of the LCS users were thinking LCS use improved their blood glucose control however 81% reported having problems using LCS. The most common problems were not liking the taste and the cost. Some (63%) reported that they have health concerns about LCS use. The most common health-related concerns were nonspecific health-related concerns (72%), concern for cancer (36%), and concerns related to unknown chemicals (24%). Most (92%) of them reported that they did not receive any recommendation not to use LCS from a healthcare professional. Among LCS users, 53% reported that LCS improved their QOL. Most (88%) of the people that were not using LCS reported that the use of LCS would not affect their QOL, and if so, 27% were thinking use of LCS would improve the QOL.

The recent LCS use group was older and had a longer duration of diabetes (37 ± 13 vs. 33 ± 12 and 22 ± 13 vs. 19 ± 11 respectively, p < 0.01 for both) otherwise there were no differences between both groups for other demographics such as sex, race, ethnicity, education, and insurance.

There were no differences in DSMQ Score, DSMQ Glucose Management Score, DSMQ Dietary Control Score, or DSMQ Health Care Score but DSMQ Physical Activity Score was higher in no recent LCS use group compared to the recent LCS use group (5.6 ± 1.1 vs. 5.3 ± 1.1, p < 0.001). After adjusting for age, sex, diabetes duration, and diabetes complications, the significance of these scores has not changed and the DSMQ Physical Activity score remained significant (Table 2).

**Table 2:** Diabetes Self-Management Questionnaire (DSMQ) general score and detailed scores of recent LCS users and non-users with T1D. (Adjusted for age, sex, diabetes duration, and diabetes complications.)

	Recent LCS use (n = 360)	No recent LCS use (n = 172)	P-value
DSMQ Score	20.6 ± 3.7	21.1 ± 3.7	0.1335
DSMQ Glucose Management Score	8.0 ± 1.9	8.1 ± 1.8	0.4560
DSMQ Dietary Control Score	5.1 ± 1.9	5.2 ± 1.9	0.3562
DSMQ Health Care Score	8.5 ± 2.0	8.5 ± 2.0	0.6816
DSMQ Physical Activity Score	5.3 ± 1.1	5.6 ± 1.1	0.0012

There was no difference between groups for diabetic retinopathy, nephropathy, and neuropathy and coronary artery disease however recent LC use group had more hypertension and overall complications (97 (27%) vs. 26 (15%) and 194 (54%) vs. 73 (42%) respectively, p < 0.01 for both) (Table 1). The odds of any complication by recent LCS use were not significant.

**Fig. 1:**
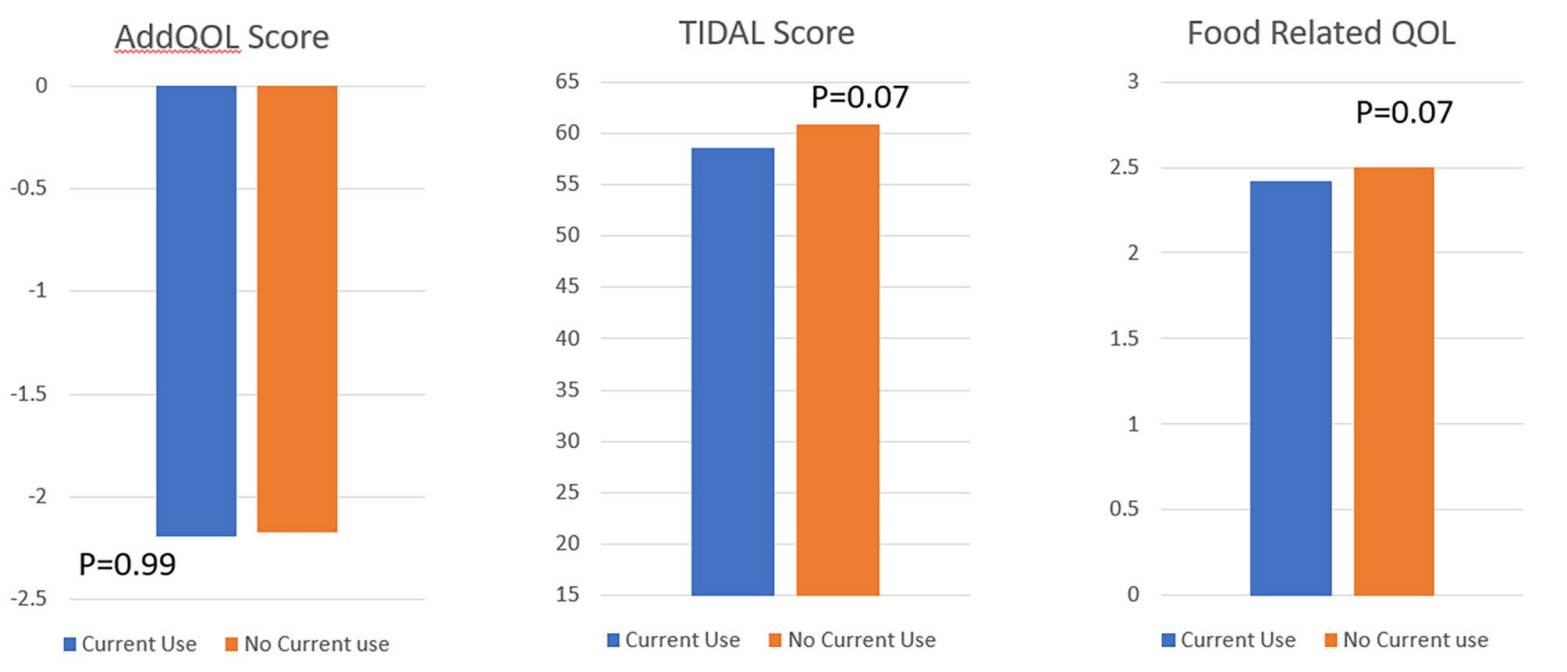
QOL assessment with different validated questionnaires by the use of LCS. (Adjusted for age, sex, diabetes duration, daily breakfast, eating outside of the home, trying to lose weight and complications.)

There was no difference in ADDQOL Score (-24.1 ± 17.1 vs. -27.4 ± 19.0, p:0.057) and Food QOL Score (2.6 ± 0.5 vs. 2.4 ± 2.4, p:0.009) between groups, but T1DAL Score was higher in the no recent LCS use group (64.6 ± 15.2 vs. 60.8 ± 15.2, p < 0.001). However, the T1DAL score was not significant between the two groups after adjusting for age, sex, diabetes duration, daily breakfast, eating outside of the home, trying to lose weight, and complications (Figure 1).

## Discussion

To our knowledge, this is the first study to evaluate the relationship between LCS use and QOL in adults with T1D. Most adults with T1D in our survey were aware of LCS and over two-thirds had used it last month. The most common use of LCS was in drinks and most users reported using it to meet glycemic targets and improve their QOL. However, recent LCS users were similar to non-users in terms of A1c, and diabetes self-management but had lower scores on physical activity-related diabetes management. A lower QOL score on the T1DAL questionnaire among recent users of LCS was attenuated to non-significant when adjusted for diabetes complications and poor dietary practices. Thus, there was no difference between recent users and non-users in terms of QOL. There was a disparity between expected glycemic and QOL improvement and QOL evaluation in the validated questionnaires. This highlights the need for healthcare professionals to provide guidance and recommendations to people with T1D who are considering using LCS with the expectation of an improvement in their QOL. Therefore, more research is needed to fully understand the relationship between LCS use and QOL of people with T1D.

Previous studies have shown conflicting results on the impact of LCS on obesity, type 2 diabetes, and cardiovascular disease. Some studies suggest a link between regular consumption of LCS and an increased risk of these conditions [13] while other studies have found no association or improvement between LCS use and cardiometabolic health outcomes [7, 14, 15]. Increased risk for hypertension in adult LCS users was also reported in previous observational studies and meta-analyses [13]. In our study, people with T1D who reported using LCS in the last month were older and had longer diabetes duration and more complications (hypertension or any complication) than those who reported no recent LCS use. However, the odds of any complication by LCS use were not significant.

In terms of glycemic management, randomized controlled studies with type 2 diabetes patients showed no significant effect on glycemic control while observational studies showed a slight association between LCS use and T2D [7]. Similarly, our study showed no difference in A1c between recent users and non-users, however, most (73%) LCS users reported feeling improvement in glycemia.

The strengths of this study were the large sample size and the use of numerous validated questionnaires to assess the QOL and diabetes management of participants. Our study has some limitations. Single-center, cross-sectional study design is the main limitation, and the temporality of the exposure and outcome cannot be ascertained in cross-sectional studies. Therefore, determining the causality is difficult. Associations between the exposure and outcome can be bi-directional. Another limitation was the inability to ensure the self-reported LCS consumption of participants. Males could be underrepresented in this study as most of the respondents were female.

## Conclusion

This is the first study that investigated LCS use and its effect on QOL in adults with T1D. Although participants reported improvement in glycemia by LCS use, QOL or glycemic profile did not differ between recent LCS users and non-users. The disparity between expected and observed results in QOL and glycemic measurements requires the guidance of healthcare professionals to clarify the benefits and barriers of LCS use.

## Data Availability

Data is available upon request from the corresponding author.

## References

[1] Akturk HK, Rompicherla S, Rioles N, et al. Factors Associated with Improved A1C among adults with type 1 diabetes in the United States. Clin Diabetes. 2022 Winter;41(1):76–80. 10.2337/cd22-0067 PMC9845074 36714244

[2] Akturk HK, Rewers A, Joseph H, Schneider N, Garg SK. Possible Ways to improve postprandial glucose control in type 1 diabetes. Diabetes Technol Ther. 2018 Jun;20(S2):224–S232. 10.1089/dia.2018.0114 29916737

[3] Chiavaroli L, Lee D, Ahmed A, et al. Effect of low glycaemic index or load dietary patterns on glycaemic control and cardiometabolic risk factors in diabetes: systematic review and meta-analysis of randomised controlled trials. BMJ. 2021 Aug;4:374:n1651. 10.1136/bmj.n1651 PMC8336013 34348965

[4] Dyson PA, Twenefour D, Breen C, et al. Diabetes UK evidence-based nutrition guidelines for the prevention and management of diabetes. Diabet Med. 2018 May;35(5):541–7. 10.1111/dme.13603 29443421

[5] U.S. Food and Drug Administration. High-intensity sweeteners. 2017. https://www.fda.gov/food/food-additives-petitions/high-intensity-sweeteners. Accessed February 9, 2023.

[6] U.S. Food and Drug Administration. Additional information about high-intensity sweeteners permitted for use in food in the United States. 2018. https://www.fda.gov/food/ingredientspackaginglabeling/foodadditivesingredients/ucm397725.htm. Accessed February 9, 2023.

[7] Miller PE, Perez V. Low-calorie sweeteners and body weight and composition: a meta-analysis of randomized controlled trials and prospective cohort studies. Am J Clin Nutr. 2014 Sep;100(3):765–77. 10.3945/ajcn.113.082826 PMC4135487 24944060

[8] Guadagnoli L, Mutlu EA, Doerfler B, Ibrahim A, Brenner D, Taft TH. Food-related quality of life in patients with inflammatory bowel disease and irritable bowel syndrome. Qual Life Res. 2019 Aug;28(8):2195–205. 10.1007/s11136-019-02170-4 PMC6625837 30900206

[9] Schmitt A, Gahr A, Hermanns N, Kulzer B, Huber J, Haak T. The diabetes self-management questionnaire (DSMQ): development and evaluation of an instrument to assess diabetes self-care activities associated with glycaemic control. Health Qual Life Outcomes. 2013 Aug;13:11:138. 10.1186/1477-7525-11-138 PMC3751743 23937988

[10] Harvard Willett Food Frequency Questionnaire. https://snaped.fns.usda.gov/library/materials/harvard-willett-food-frequency-questionnaire. Accessed 8 Feb 2023.

[11] Bąk E, Nowak-Kapusta Z, Dobrzyn-Matusiak D, Marcisz-Dyla E, Marcisz C, Krzemińska SA. An assessment of diabetes-dependent quality of life (ADDQoL) in women and men in Poland with type 1 and type 2 diabetes. Ann Agric Environ Med. 2019 Sep;19(3):429–38. 10.26444/aaem/99959 31559799

[12] Hilliard ME, Minard CG, Marrero DG, et al. Health-related quality of life in parents and partners of people with type 1 diabetes: development and validation of type 1 diabetes and life (T1DAL) measures. Fam Syst Health. 2021 Jun;39(2):234–47. 10.1037/fsh0000507 PMC8376806 33900103

[13] Azad MB, Abou-Setta AM, Chauhan BF et al. Nonnutritive sweeteners and cardiometabolic health: a systematic review and meta-analysis of randomized controlled trials and prospective cohort studies. CMAJ 2017 Jul 17;189(28):E929–39. 10.1503/cmaj.161390 PMC5515645 28716847

[14] Brown RJ, de Banate MA, Rother KI. Artificial sweeteners: a systematic review of metabolic effects in youth. Int J Pediatr Obes. 2010 Aug;5(4):305–12. 10.3109/17477160903497027 PMC2951976 20078374

[15] Lohner S, Kuellenberg de Gaudry D, Toews I, Ferenci T, Meerpohl JJ. Non-nutritive sweeteners for diabetes mellitus. Cochrane Database Syst Rev 2020 May 25;5(5):CD012885. 10.1002/14651858.CD012885.pub2 PMC7387865 32449201

